# Collagenofibrotic Glomerulopathy With Concurrent Ostium Secundum Atrial Septal Defect and Anti-Ku Antibody Positivity: A Rare Case

**DOI:** 10.7759/cureus.112641

**Published:** 2026-07-14

**Authors:** Shreyas Thakare, Shilpa S Kuthe, Fahad Idrees Shaikh, Nishka Tiwari, Zainab

**Affiliations:** 1 Internal Medicine, N.K.P. Salve Institute of Medical Sciences and Research Centre and Lata Mangeshkar Hospital, Nagpur, IND; 2 General Medicine, N.K.P. Salve Institute of Medical Sciences and Research Centre and Lata Mangeshkar Hospital, Nagpur, IND; 3 Medical Education, N.K.P. Salve Institute of Medical Sciences and Research Centre and Lata Mangeshkar Hospital, Nagpur, IND; 4 Medicine and Surgery, Dr VRK Women’s Medical College, Hyderabad, IND

**Keywords:** anti-ku antibody, atrial septal defect, collagenofibrotic glomerulopathy, immunohistochemistry, nephrotic syndrome, renal biopsy, type iii collagen

## Abstract

Collagenofibrotic glomerulopathy (CG) is an exceptionally rare idiopathic glomerular disease defined by pathological intraglomerular accumulation of type III collagen fibrils within the mesangial matrix and subendothelial space. We report the case of a 35-year-old woman presenting with generalized anasarca, frothy urine, and exertional dyspnea. Laboratory investigations revealed nephrotic syndrome with a 24-hour urinary protein of 3,658 mg/day, serum albumin of 2 g/dL, and progressive renal dysfunction with serum creatinine rising from 3.13 to 5.25 mg/dL. Echocardiography incidentally demonstrated an ostium secundum atrial septal defect with mild pulmonary arterial hypertension, while serology showed a strongly positive anti-Ku antibody (3+). Renal biopsy also showed diffuse mesangial expansion by Congo red-negative eosinophilic material with marked narrowing and focal obliteration of capillary lumina. Immunofluorescence studies demonstrated weak mesangial staining for both kappa and lambda light chains. Immunohistochemistry confirmed faint positive staining for type III collagen in mesangial regions, confirming the diagnosis of CG. Treatment consisted of diuretics, antihypertensive therapy, corticosteroids, and supportive measures. Despite these interventions, renal function continued to deteriorate, with estimated glomerular filtration rate decreasing from 19 to 10 mL/minute/1.73 m² at discharge. The present case highlights the utility of immunohistochemistry in establishing the diagnosis of CG when electron microscopy is unavailable.

## Introduction

Collagenofibrotic glomerulopathy (CG), also termed collagen type III glomerulopathy, is an extremely rare idiopathic renal disease first described in 1979 by Arakawa et al. from Japan [1]. It is characterized by abnormal intraglomerular deposition of type III collagen fibrils within the mesangial matrix and subendothelial space, accompanied by markedly elevated serum procollagen type III peptide (PIIINP) levels [1]. Type III collagen is a ubiquitous extracellular structural protein normally confined to the renal interstitium and blood vessel walls; its intraglomerular presence is strictly pathological [2]. Initially considered a skeletal deformity-free variant of nail-patella syndrome due to similar glomerular collagen deposition, CG was recognized as a distinct clinicopathological entity in the 1990s and was incorporated into formal glomerular disease classifications [2]. Interleukin-4 (IL-4) selectively stimulates type III collagen synthesis in human glomerular cells, and IL-4-neutralizing antibodies have been shown to prevent glomerulosclerosis in IL-4-transgenic mice, implicating a cytokine-mediated local renal mechanism [3]. Serum PIIINP and hyaluronan levels have been observed to normalize following renal transplantation, further supporting an intrarenal origin [3].

Approximately 100 cases have been reported globally, predominantly from Japan, with the disease manifesting as either a sporadic adult-onset form or a familial childhood-onset form with autosomal recessive inheritance [4]. Clinically, patients present with nephrotic-range proteinuria, peripheral edema, hypertension, and progressive renal insufficiency with potential rapid progression to end-stage renal disease (ESRD) [4]. At present, no targeted treatment is available. Management is largely supportive and commonly includes blockade of the renin-angiotensin system to reduce proteinuria and slow disease progression [4]. Diagnosis requires a renal biopsy; light microscopy reveals diffuse mesangial expansion with eosinophilic, Congo red-negative material, typically with negative immunofluorescence [5]. Electron microscopy (EM) provides definitive confirmation by demonstrating characteristic banded type III collagen fibrils measuring 50-100 nm in diameter with long-spacing striations of 25-30 nm, findings that may be supported by type III collagen immunohistochemistry (IHC) [5]. In resource-limited settings where EM is unavailable, IHC for type III collagen serves as an important confirmatory tool [5]. We present the case of a 35-year-old woman with IHC-confirmed CG alongside an incidentally discovered ostium secundum atrial septal defect (ASD), mild pulmonary arterial hypertension, and a strongly positive anti-Ku antibody, a combination not previously described in the literature.

## Case presentation

A 35-year-old female presented to the Department of Medicine with a three-month history of generalized swelling, initially periorbital and facial, subsequently extending to the bilateral lower limbs and trunk, accompanied by frothy urine for two months and progressive exertional dyspnea for the same duration. There was no history of fever, hematuria, dysuria, oliguria, joint pain, skin rash, oral ulcers, alopecia, jaundice, or significant weight loss. She had been diagnosed with systemic hypertension at the age of 30 years and reported irregular adherence to antihypertensive therapy. Family history revealed hypertension in both parents. Obstetric history was G2P1A1. There was no history of diabetes mellitus, thyroid disorder, or non-steroidal anti-inflammatory drug use.

**Figure 1:**
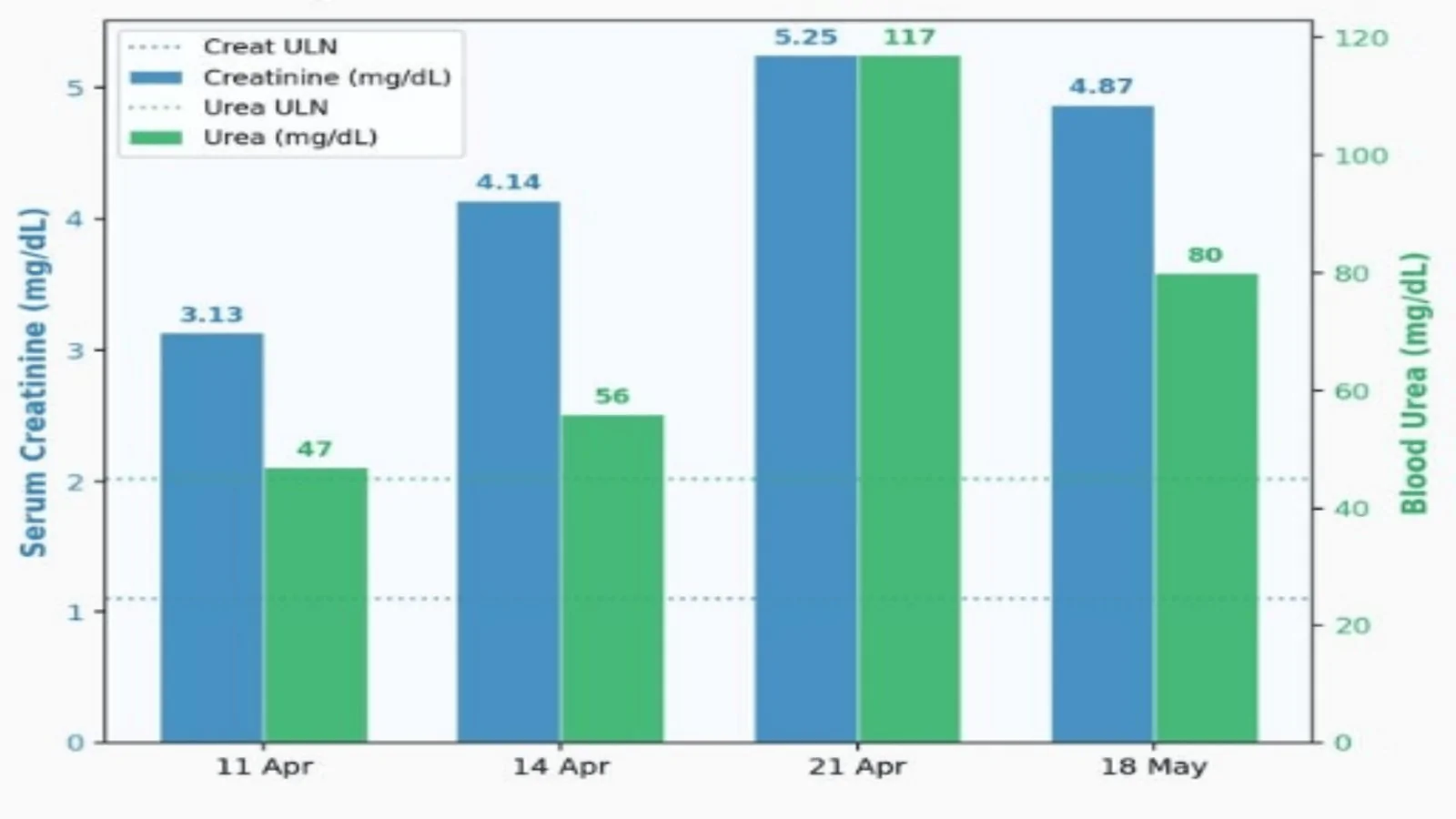
Serial creatinine and urea trend.

The patient was conscious, oriented, and alert. Vital signs showed a pulse rate of 110 beats/minute, blood pressure of 180/100 mmHg, respiratory rate of 22 breaths/minute, and SpO₂ of 98% on room air. Pallor was present. Severe bilateral pitting edema extending to the thighs (Grade 4), periorbital and facial puffiness, and edema of the upper limbs and abdomen were noted. Cardiovascular examination revealed wide splitting of the second heart sound. Respiratory examination demonstrated absent air entry in the right intrascapular region with vesicular breath sounds elsewhere. No significant abnormalities were detected on abdominal or neurological examination. Initial hematological evaluation showed anemia, with a hemoglobin level of 7.5 g/dL. Peripheral smear examination demonstrated mild hypochromia, anisopoikilocytosis, occasional microcytes, elliptocytes, and teardrop cells, suggesting a possible microangiopathic process. Renal function deteriorated progressively: serum creatinine rose from 3.13 mg/dL on admission (April 11) to 5.25 mg/dL by April 21, with blood urea increasing from 47 to 117 mg/dL, and the serial biochemical investigations demonstrated worsening renal function. Serum creatinine increased from 3.13 mg/dL at admission on April 11 to 5.25 mg/dL by April 21. Blood urea levels also rose from 47 mg/dL to 117 mg/dL during the same period. At the follow-up visit on May 18, serum creatinine had decreased slightly to 4.87 mg/dL, while blood urea measured 80 mg/dL. Electrolyte levels and liver function parameters were consistently within the normal range during the course of evaluation. Detailed laboratory findings are provided in Table 1, whereas trends in renal function over time are shown in Table 2. The serial urea-creatinine trend is illustrated in Figure 1.

**Table 1:** Laboratory investigations on admission. MCV = mean corpuscular volume; PCR = protein-to-creatinine ratio; RBC = red blood cell; ANA = antinuclear antibody; ANCA = antineutrophil cytoplasmic antibody

Parameter	Result	Reference range
Hemoglobin	7.5 g/dL	12–15 g/dL (F)
Total leucocyte count	4260 cells/µL	4000–11000 cells/µL
Hematocrit	27%	36–46%
Platelets	173,000/µL	150,000–400,000/µL
MCV	87.7 fL	80–100 fL
Peripheral smear	Mild hypochromia, anisopoikilocytosis, microcytes, elliptocytes, occasional teardrop cells	—
Serum creatinine (admission 11/04)	3.13 mg/dL	0.5–1.1 mg/dL
Blood urea (admission 11/04)	47 mg/dL	15–45 mg/dL
Serum sodium	136 mEq/L	135–145 mEq/L
Serum potassium	3.7 mEq/L	3.5–5.0 mEq/L
Serum albumin	2.0 g/dL	3.5–5.0 g/dL
Spot urine PCR	7.8	<0.2 (normal)
24-hour urinary protein	3,658 mg/day	<150 mg/day
Urine routine, protein	4+	Nil (Negative)
Urine routine, RBC/pus cells/casts	RBC 4/HPF; pus cells/casts nil	RBC 0–2/HPF; pus cells 0–5/HPF; casts absent
Serum calcium	7.9 mg/dL	8.5–10.5 mg/dL
Serum phosphate	5.6 mg/dL	2.5–4.5 mg/dL
Serum uric acid	6.7 mg/dL	2.4–6.0 mg/dL
Serum magnesium	1.7 mg/dL	1.7–2.2 mg/dL
Complement C3	119 mg/dL	90–180 mg/dL
Complement C4	28.8 mg/dL	16–47 mg/dL
ANA (immunofluorescence)	1:100 weakly positive, nuclear nucleolar pattern	Negative
Anti-Ku antibody (ANA blot)	3+ strongly positive	Negative
P-ANCA/c-ANCA	Negative	Negative
Liver function tests	Within normal limits	—

**Table 2:** Serial renal function and hematology (at admission and at follow-up). eGFR = estimated glomerular filtration rate

Parameter	Reference range	April 11	April 14	April 21	May 18
Serum creatinine (mg/dL)	0.5–1.1	3.13	4.14	5.25	4.87
Blood urea (mg/dL)	15–45	47	56	117	80
Sodium (mEq/L)	135–145	136	135	136	139
Potassium (mEq/L)	3.5–5.0	3.7	3.9	4.5	4.2
Hemoglobin (g/dL)	12–15 (female)	7.5	8.4	—	—
eGFR (mL/minute/1.73 m²)	>90	~19	—	~10	~12

**Figure 2:**
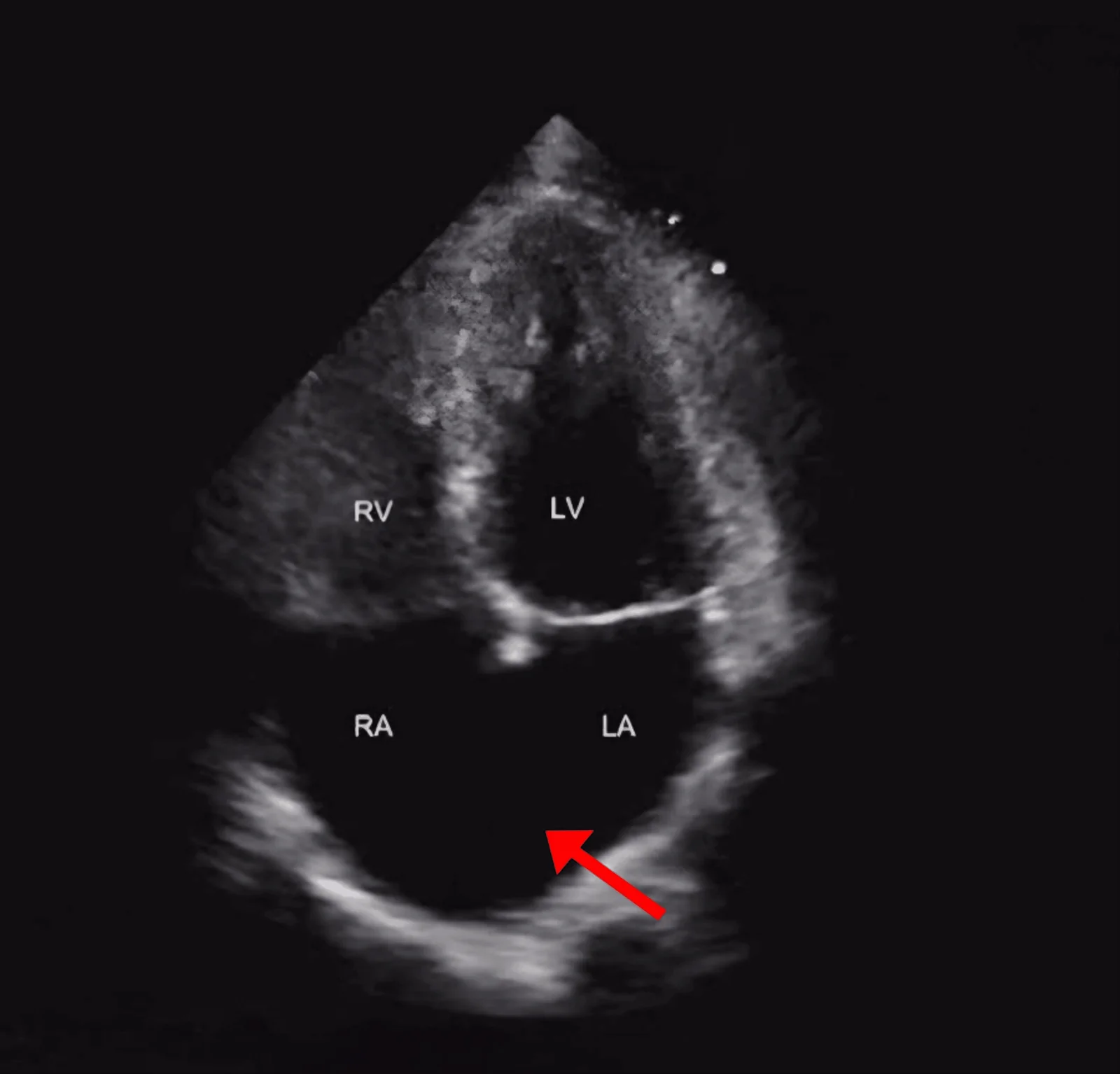
Echocardiography: ostium secundum ASD. Two-dimensional echocardiography apical four-chamber view (RV, LV, RA, and LA): 1.3 cm ostium secundum ASD with color Doppler left-to-right shunt. The red arrow indicates the site of the ostium secundum ASD. ASD = atrial septal defect; RV = right ventricle; LV = left ventricle; RA = right atrium; LA = left atrium

Estimated glomerular filtration rate (eGFR) declined from ~19 mL/minute/1.73 m² on admission to ~10 mL/minute/1.73 m² at discharge. Urinalysis showed 4+ proteinuria with red blood cells 4/hpf, without pus cells or casts. The spot urine protein-to-creatinine ratio was 7.8, and the 24-hour urinary protein was 3,658 mg/day. Serum albumin was 2.0 g/dL, confirming nephrotic syndrome. Serum calcium was 7.9 mg/dL, phosphate 5.6 mg/dL, uric acid 6.7 mg/dL, and magnesium 1.7 mg/dL. On immunological workup, antinuclear antibody (ANA) by immunofluorescence was weakly positive at 1:100 with a nuclear nucleolar pattern; the ANA blot demonstrated anti-Ku antibody strongly positive (3+). P-antineutrophil cytoplasmic antibody (ANCA) and c-ANCA were negative. Complement levels were normal (C3: 119 mg/dL, C4: 28.8 mg/dL). Ultrasonography revealed bilateral echogenic kidneys with poor corticomedullary differentiation, minimal ascites, and mild right pleural effusion. Two-dimensional echocardiography demonstrated an ostium secundum ASD of 1.3 cm with left-to-right shunting and pulmonary artery systolic pressure of 39 mmHg, indicating mild pulmonary arterial hypertension. Colour Doppler imaging was not captured digitally; the ASD with a left-to-right shunt and pulmonary artery systolic pressure of 39 mmHg were documented in the formal echocardiography report (Figure 2).

**Figure 3:**
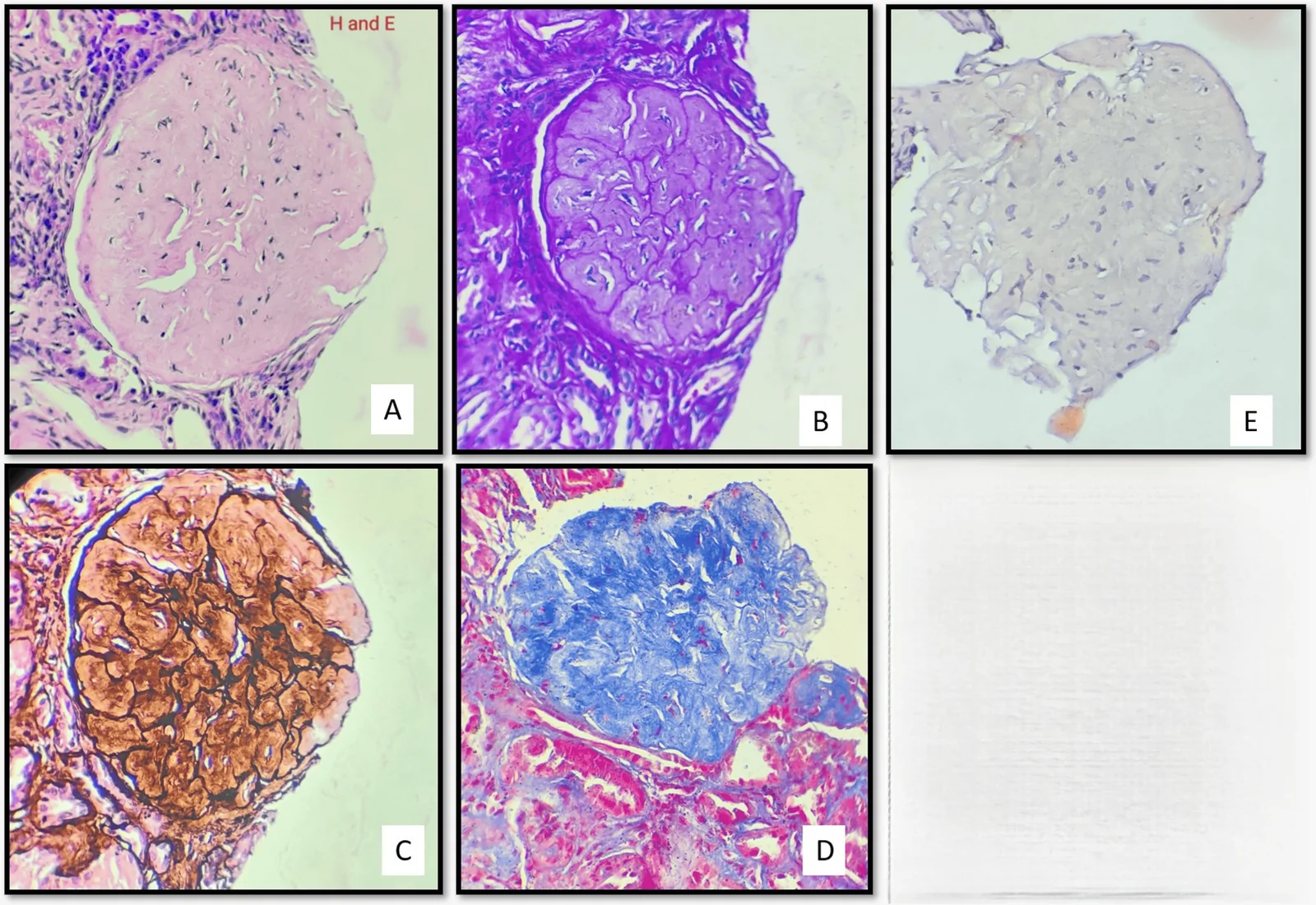
Renal biopsy: light microscopic findings demonstrating diffuse mesangial expansion by eosinophilic material showing variable staining characteristics consistent with collagenofibrotic glomerulopathy (all microphotographs ×40). (A) Hematoxylin and eosin stain showing pale eosinophilic mesangial deposits. (B) Periodic acid-Schiff (PAS) stain demonstrating pale PAS-positive deposits. (C) Jones methenamine silver stain showing silver-negative deposits. (D) Masson trichrome stain demonstrating blue-staining deposits. (E) Congo red stain negative for amyloid deposition.

Renal biopsy showed enlarged glomeruli with diffuse, extensive mesangial expansion by hyaline, pale eosinophilic material. This material was periodic acid-Schiff-pale, variably silver-staining, pale blue on Masson trichrome, and Congo red-negative, with complete luminal obliteration. No mesangial or endocapillary hypercellularity, duplication, spikes, eyelash sign, pinholes, wire-loop lesions, hyaline thrombi, segmental necrosis, crescents, or features of thrombotic microangiopathy were identified. The tubulointerstitium showed acute tubular injury with focal cytoplasmic vacuolation, occasional hyaline and granular casts, patchy lymphomononuclear interstitial infiltrate, focal foam cell aggregates, and patchy tubular atrophy with interstitial fibrosis involving approximately 20% of the sampled cortex. Focal arteriolar hyalinosis was present, as shown in Figure 3.

**Figure 4:**
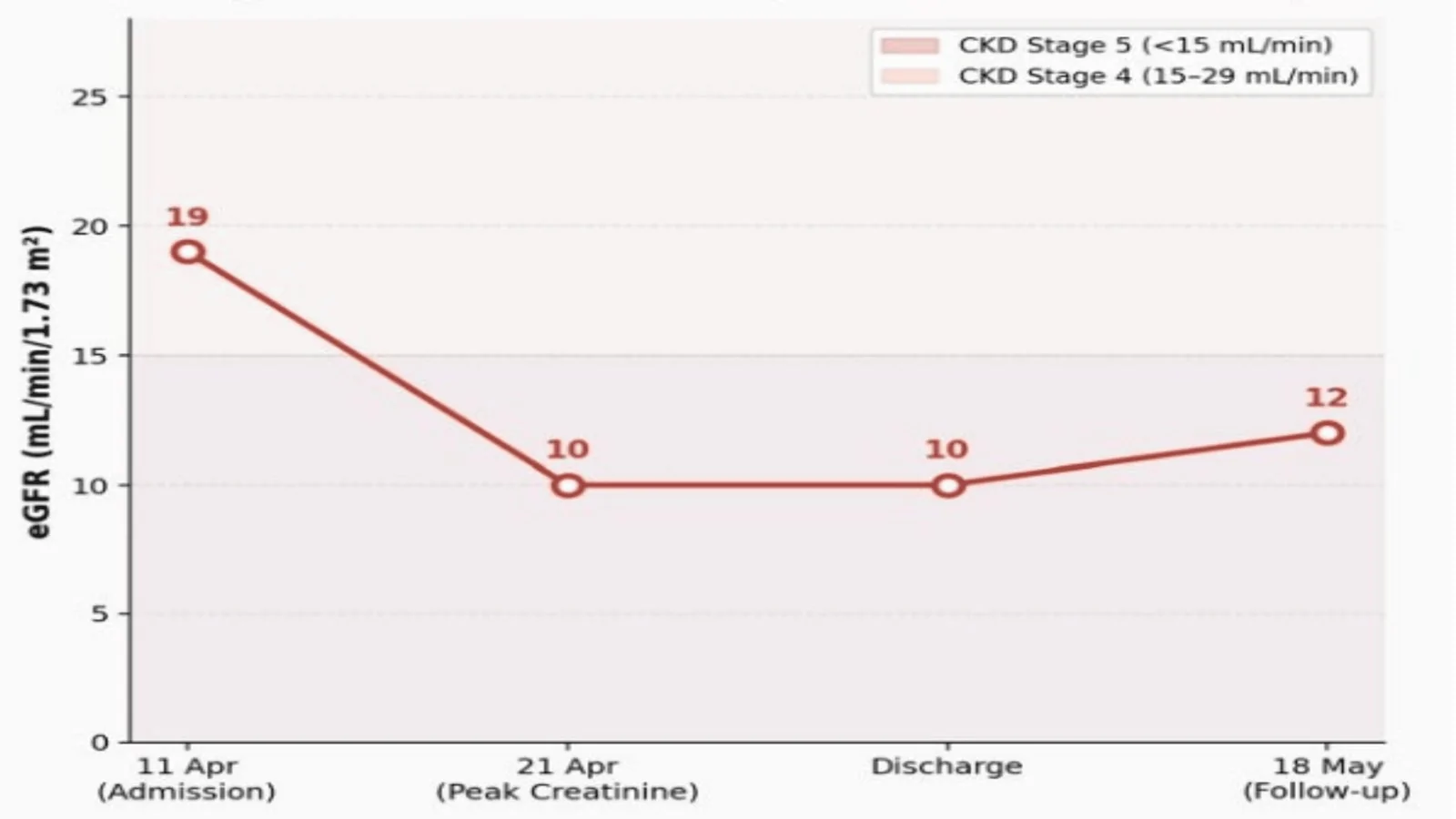
eGFR trend graph during admission and follow-up (CKD-EPI equation). A rapid decline from approximately 19 to 10 mL/minute/1.73 m² was observed during admission, with marginal stabilization at the one-month follow-up. X-axis: time point. Y-axis: eGFR (mL/minute/1.73 m²). eGFR = estimated glomerular filtration rate; CKD = chronic kidney disease

Immunofluorescence photomicrographs were not available for reproduction; findings are as reported by the referring pathologist. Faint mesangial kappa and lambda light chain deposits were seen, with IgG, IgA, IgM, C3, and C1q being negative. Given the characteristic light microscopy findings and the non-availability of EM at the primary reporting center, the biopsy block was referred to the Department of Laboratory Medicine (Histopathology and Cytology) at Sir H.N. Reliance Foundation Hospital and Research Centre, Mumbai, for IHC. IHC using a single antibody against type III collagen (Ventana platform) on paraffin-embedded tissue demonstrated faint positive staining in a few mesangial regions. This finding, in conjunction with the light microscopy pattern and the clinical presentation, confirmed the diagnosis of collagenofibrotic glomerulopathy. The patient was managed with aggressive diuresis using intravenous torsemide and oral metolazone, antihypertensive therapy with olmesartan and clonidine, high-dose corticosteroids, protein supplementation, and one unit of packed red blood cells for symptomatic anemia. Despite symptomatic improvement in edema and dyspnea, renal function continued to decline: eGFR fell from approximately 19 mL/minute/1.73 m² on admission to 10 mL/minute/1.73 m² at discharge, as shown in Table 3 and Figure 4. The patient was discharged with close nephrology follow-up and was counselled regarding the risk of progression to ESRD.

**Table 3:** eGFR trend from admission to follow-up. eGFR = estimated glomerular filtration rate

Time point	eGFR (mL/minute/1.73 m²)
April 11: Admission	~19
April 21: Peak creatinine	~10
Discharge	10
May 18: Follow-up	~12

## Discussion

CG is an exceptionally rare cause of nephrotic syndrome, with fewer than 100 confirmed cases reported globally, predominantly from Japan and other Asian countries [6]. Reports from the Indian subcontinent remain sparse, and the present case adds to this limited pool, while also documenting novel concurrent findings not previously described in association with CG [6]. The histological differential diagnosis of diffuse mesangial expansion by Congo red-negative eosinophilic material encompasses several entities that must be systematically excluded [7]. Amyloidosis is excluded by Congo red negativity. Fibrillary glomerulonephritis (FGN) and immunotactoid glomerulopathy (ITG) both demonstrate immunoglobulin and C3 deposits on immunofluorescence, which were absent as dominant deposits in the present case [7]. On EM, FGN shows randomly arranged 10-30 nm fibrils, whereas ITG demonstrates larger microtubular deposits in organized arrays; CG is distinguished by banded type III collagen fibrils of 50-100 nm diameter with characteristic 25-30 nm cross-striations [7]. Fibronectin glomerulopathy shows negative immunofluorescence but is identified by fibronectin immunostaining; nail-patella syndrome is excluded by *LMX1B* gene mutation analysis, and the absence of skeletal and nail abnormalities; and diabetic nodular glomerulosclerosis is differentiated by clinical context and EM findings [8]. In resource-limited settings where EM is unavailable, IHC for type III collagen on paraffin-embedded tissue has been validated as an alternative confirmatory test [9]. In the present case, faint but definite mesangial positivity for type III collagen on IHC (Ventana platform, CAP-accredited laboratory) established the diagnosis in the absence of EM. The faintness of staining may reflect early or focal collagen deposition and does not negate the diagnosis when the clinical and light microscopy contexts are characteristic, as has been documented in prior case series [9]. The differential diagnosis and comparison with previously reported Indian cases are summarized in Table 4 and Table 5, respectively.

**Table 4:** Differential diagnosis of IF-negative mesangial deposition (Congo red-negative, IF-negative glomerular deposition) diseases. IF = immunofluorescence; PAS = periodic acid-Schiff; EM = electron microscopy; GN = glomerulonephritis; IHC = immunohistochemistry; GBM = glomerular basement membrane; KW = Kimmelstiel-Wilson; DNAJB9 = DnaJ homolog subfamily B member 9

Entity	Congo red	IF	EM findings	PAS nodules	Distinguishing feature
Collagenofibrotic glomerulopathy [7-9]	Negative	Faint kappa/lambda (present case); typically negative	Banded type III collagen 50–100 nm, 25–30 nm striations	Negative	Type III collagen IHC positive (present case: faint +)
Amyloidosis [7]	Positive	Negative	Straight non-banded fibrils 8–12 nm	Negative	Congo red positive; apple-green birefringence
Fibrillary GN [7,10]	Negative	IgG, C3 positive	Random fibrils 10–30 nm	Negative	DNAJB9 IHC positive
Immunotactoid GN [7,10]	Negative	Monoclonal IgG, C3	Microtubules >30 nm in parallel arrays	Negative	Monoclonal Ig; paraprotein workup
Fibronectin GN [8]	Negative	Negative	Short vague fibrils	Negative	Fibronectin IHC positive
Nail-patella syndrome [8]	Negative	Negative	Irregular collagen in GBM with lucent areas	Negative	LMX1B mutation; skeletal/nail abnormalities
Diabetic nodular GS [7]	Negative	Negative	No organized fibrils; GBM thickening	Present (KW nodules)	Diabetes history; EM context

**Table 5:** Comparison with previously reported Indian cases of collagenofibrotic glomerulopathy. NS = nephrotic syndrome; HTN = hypertension; AKI = acute kidney injury; CKD = chronic kidney disease; EM = electron microscopy; IHC = immunohistochemistry; PIIINP = procollagen type III peptide; OLM = olmesartan; ESRD = end-stage renal disease

Author/Year	Age/Sex	Presentation	Diagnostic tools	PIIINP	Treatment	Outcome
Patro et al., 2011 [3]	35/Female	NS, HTN	LM + IF; no EM	Not done	Steroids, supportive	Partial remission
Dhanapriya et al., 2017 [8]	28/Male	NS, AKI	LM + IF; EM done	Elevated	Supportive	ESRD at 2 yrs
Modi et al., 2020 [6]	42/Female	NS, CKD	LM + IF + IHC	Not done	ACEi, supportive	Progressive CKD
Matthai et al., 2020 [9]	50/Male	NS, HTN	LM + IF + EM	Not reported	Supportive	Stable at follow-up
Present case, 2026	35/Female	NS, HTN, ASD, anti-Ku+	LM + IF + IHC (no EM)	Not available	Steroids, OLM, diuretics	eGFR 19→10; follow-up ongoing

A notable finding in the present case was faint mesangial kappa and lambda light chain deposition on immunofluorescence, without significant IgG, IgA, IgM, C3, or C1q deposits. This pattern of low-level polyclonal light chain trapping in mesangial deposits has been described as an incidental finding in non-immune glomerular deposition diseases and does not satisfy the diagnostic criteria for light-chain deposition disease or amyloidosis [10]. The Congo red negativity and the IHC-confirmed type III collagen staining in the present case confirm that the light chain deposits are not pathogenetically dominant and should be interpreted as a secondary trapping phenomenon within the abnormal collagenous mesangial matrix [10].

The pathogenesis of CG remains incompletely understood. Both renal-limited and systemic forms involving extrarenal collagen deposition have been described, and debate persists regarding its classification as a primary renal or systemic disease [11]. Cases with accumulation of both type I and type III collagen in the glomeruli have been documented, suggesting that the spectrum of aberrant collagen deposition may be broader than originally characterized [11]. An association between inherited factor H deficiency and CG has been described, raising the possibility that complement-mediated glomerular endothelial injury may facilitate aberrant subendothelial collagen access [12]. In the present patient, complement levels were normal (C3: 119 mg/dL, C4: 28.8 mg/dL), making factor H deficiency less likely as a pathogenic mechanism; however, a formal factor H assay was not performed [12]. The co-occurrence of an ostium secundum ASD (1.3 cm, left-to-right shunt, pulmonary artery systolic pressure: 39 mmHg) represents, to our knowledge, an undescribed association with CG. Given the established role of type III collagen in cardiac extracellular matrix remodelling and the debate regarding CG as a potential systemic collagen disorder, a biologically plausible hypothesis exists that aberrant type III collagen metabolism could contribute to structural cardiac defects in genetically predisposed individuals [13]. This finding warrants documentation and prospective evaluation in future reported cases.

The strongly positive anti-Ku antibody (3+) with weak ANA (1:100 nucleolar) in the absence of dominant immune deposits on biopsy and with normal complement levels represents another unreported finding in CG. Anti-Ku antibodies are rare autoantibodies associated with connective tissue diseases, with renal involvement reported in up to 12.8% of anti-Ku-positive patients [14]. The biopsy findings in our patient were inconsistent with lupus nephritis or ANCA-associated vasculitis. Anti-Ku antibodies are rare myositis-associated autoantibodies typically identified in connective tissue disease overlap syndromes, particularly systemic sclerosis and idiopathic inflammatory myopathies. Although renal involvement has occasionally been reported in anti-Ku-positive patients, no causal relationship with CG has been established. In our patient, the characteristic renal biopsy findings, normal complement levels, and absence of significant immune-complex deposition suggest that anti-Ku positivity is more likely an incidental serological finding or a marker of underlying autoimmune predisposition than a direct pathogenic mechanism. The anti-Ku positivity may represent an epiphenomenon, an incidental connective tissue overlap, or a serological marker of a broader systemic collagen dysmetabolism. Systematic autoantibody profiling in future CG cases would help clarify this association [14]. Serum anti-PLA2R antibody testing was not performed. Although it forms part of the evaluation of nephrotic syndrome, the characteristic histopathological findings confirmed CG, and there was no morphological evidence suggestive of membranous nephropathy.

Management of CG remains entirely supportive. High-dose corticosteroids were administered empirically, given the acute renal deterioration and anti-Ku antibody positivity suggesting a possible immune-mediated component; however, their efficacy in CG is unestablished, and this decision was made with this limitation explicitly acknowledged. The decline of eGFR from 19 to 10 mL/minute/1.73 m² during admission underscores the aggressive trajectory that CG can follow once advanced renal impairment is established, and early preparation for renal replacement therapy is essential.

## Conclusions

We present a rare case of IHC-confirmed CG in a young woman characterized by nephrotic syndrome, progressive renal failure, faint mesangial kappa and lambda deposits on immunofluorescence, and the novel co-occurrence of an ostium secundum ASD with pulmonary hypertension and strongly positive anti-Ku antibody. IHC for type III collagen performed at a CAP-accredited laboratory confirmed the diagnosis in the absence of EM. This case demonstrates that IHC is a viable confirmatory tool in resource-limited settings, highlights the importance of systematic cardiac evaluation and autoantibody profiling in CG workup, and adds two previously unreported associations, namely, structural cardiac anomaly and anti-Ku antibody positivity, to the literature on this rare entity.
